# Absence of Parallel Fibre to Purkinje Cell LTD During Eyeblink Conditioning

**DOI:** 10.1038/s41598-018-32791-7

**Published:** 2018-10-03

**Authors:** Fredrik Johansson, Dan-Anders Jirenhed, Anders Rasmussen, Riccardo Zucca, Germund Hesslow

**Affiliations:** 1 Associative learning group, Department of Experimental Medical Science, Lund University, Lund, Sweden; 2 The Linnaeus Center Thinking in Time: Cognition, Communication & Learning, Lund University, Lund, Sweden; 3 Laboratory for Synthetic Perceptive, Emotive, and Cognitive Systems, Department of Information and Communications Technologies, Universitat Pompeu Fabra, Barcelona, Spain

## Abstract

Long-term depression (LTD) of parallel fibre/Purkinje cell synapses has been the favoured explanation for cerebellar motor learning such as classical eyeblink conditioning. Previous evidence against this interpretation has been contested. Here we wanted to test whether a classical conditioning protocol causes LTD. We applied a conditioning protocol, using a train of electrical pulses to the parallel fibres as the conditional stimulus. In order to rule out indirect effects caused by antidromic granule cell activation or output from Purkinje cells that might produce changes in Purkinje cell responsiveness, we focused the analysis on the first pulse in the conditional stimulus, that is, before any indirect effects would have time to occur. Purkinje cells learned to respond with a firing pause to the conditional stimulus. Yet, there was no depression of parallel fibre excitation after training.

## Introduction

The textbook view of motor learning in the cerebellum is that it mainly involves long-term depression (LTD) of parallel fibre to Purkinje cell (pf/Pc) synapses^1–3^ and it has been widely assumed that eyeblink conditioning, a form of cerebellar motor learning, depends on LTD^4–6^. This view has recently been questioned because it cannot readily account for timing of conditional responses^7–9^ and because knockout mice with impaired LTD and rats in which LTD was pharmacologically prevented could still learn conditional responses^10,11^. It is conceivable, however, that conditioning normally involves LTD but that, if LTD is blocked, other mechanisms can compensate. Indeed, recent findings have suggested the likely existence of alternative learning mechanisms^8,12,13^. It is therefore of crucial importance to determine if a classical conditioning protocol actually causes LTD.

During conditioning, the conditional stimulus (CS), carried by the mossy and parallel fibres, acquires the ability to suppress simple spike firing in the Purkinje cells^13,14^. This Purkinje cell pause response is adaptively timed and drives the overt conditional blink response (CR)^12,15^

In an experiment where the CS consisted of a train of electrical stimulus pulses to the mossy fibres in decerebrate ferrets, it was observed that, except for the CR period of simple spike suppression, each stimulus pulse elicited a simple spike in the Purkinje cell with about the same probability before and after conditioning^16^, suggesting that no LTD had occurred. Against this interpretation, it could be argued that the individual mossy fibre stimuli could have successively activated different sets of granule cells^4,5^, and that a lack of reduced responsiveness of the Purkinje cell to the CS could reflect a lack of LTD only in a subset of synapses not involved in generating the CR.

As a more stringent test of the LTD hypothesis, we have previously studied Purkinje cell responses to pf stimuli^8^ (for details of the methodology, see ref.^8^). When a train of stimuli is directed to the pfs, each stimulus will excite the same population of fibres. If a particular stimulus in the CS train has the same excitatory effect on the Purkinje cell before and after conditioning, it will indicate that no LTD had occurred. A problem with this argument is that various indirect loops, say antidromic pf activation or Purkinje cell output, could modulate granule cell activity and cause a time varying activity in the pfs. However, this would not apply to the first pulse in the CS train, because there would not be sufficient time for such loops to influence granule cells. If the classical conditioning protocol actually causes any LTD, it should be visible as a reduced probability of the first stimulus pulse to elicit a spike in the Purkinje cell. To investigate this possibility, we performed a new analysis of these experiments.

## Materials and Methods

**Figure 1:**
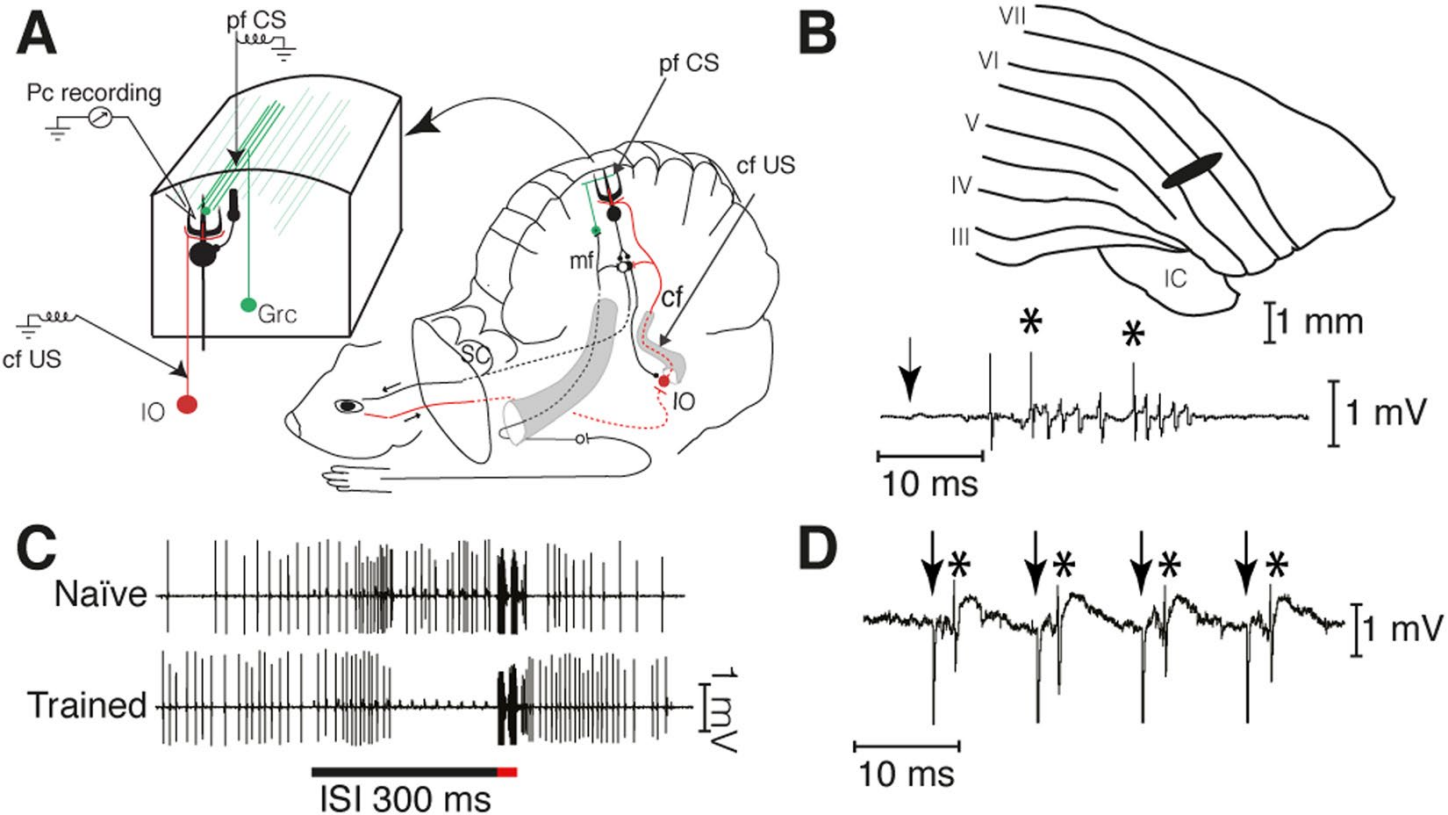
Experimental paradigm. (**A**) Sites of climbing fiber (cf US) and parallel fiber (pf CS) stimulation and Purkinje cell recording (Pc recording). IO, inferior olive; Grc, granule cell; SC, superior colliculus. (**B**) Blink-controlling area in cerebellar cortex. IC, inferior colliculus; Roman numerals indicate cerebellar lobules. Below is a single-cell recording of two complex spikes, indicated by asterisks, elicited by periocular stimulation (1 pulse, 1 mA). The arrow indicates the time of stimulation. (**C**) Typical examples of naïve and conditioned Purkinje cell responses to a forelimb conditional stimulus. Activity seen during the US period is stimulation artefacts. (**D**) Simple spikes, indicated by asterisks, elicited by pf stimulation (arrows).

All experiments were approved by the local Animal Experimentation Ethics Committee of Malmö-Lund, and all experiments were performed in accordance with relevant guidelines and regulations. Twelve male 1 year old ferrets were decerebrated and prepared for stimulation and recording in the cerebellum under anesthesia as previously described^8,17^. The experimental setup is shown in Fig. 1A. All recordings were made in a known blink-controlling area^14^ of cortical lobule HVI and each Purkinje cell was identified as controlling the eyelid by short-latency complex spikes in response to electrical periocular stimulation (Fig. 1B). When the CS consists of forelimb or mossy fiber stimulation these Purkinje cells consistently develop typical conditioned responses^14^ as shown in Fig. 1C. Here, the CS was a 100 Hz train of electrical stimuli (2–20 μA, 0.1 ms) applied directly to the parallel fibers. The location of parallel fiber stimulating electrodes was confirmed by eliciting simple spikes (Fig. 1D). The US consisted of two five-pulse 500 Hz trains of stimuli (30–400 μA, 0.1 ms) separated by 10 ms applied to ipsilateral climbing fibers. Parallel fibers and climbing fibers were stimulated with platinum tungsten electrodes (pulled and ground tips, 25 μm core diameter).

Two training protocols were used, one with a 300 ms CS, where the US was delivered 150 ms after CS onset and one with a 800 ms CS where the US was delivered 200 ms after CS onset. Using a CS that continues beyond the US serves two purposes. First, in standard protocols where the CS and US co-terminate the end of a Purkinje cell response might merely reflect the termination of the CS as opposed to reflecting learning of the interval between the CS and the US. Second, since each stimulus pulse excites the same population of parallel fibres it is of interest to assess the probability of spike elicitation beyond the CS-US interval where LTD but not conditioned responding would be expected (for references see^7^). The intertrial interval was 15 +/− 1 s (randomized). For further details concerning recording techniques and analysis, see^8,17^.

## Results

**Figure 2:**
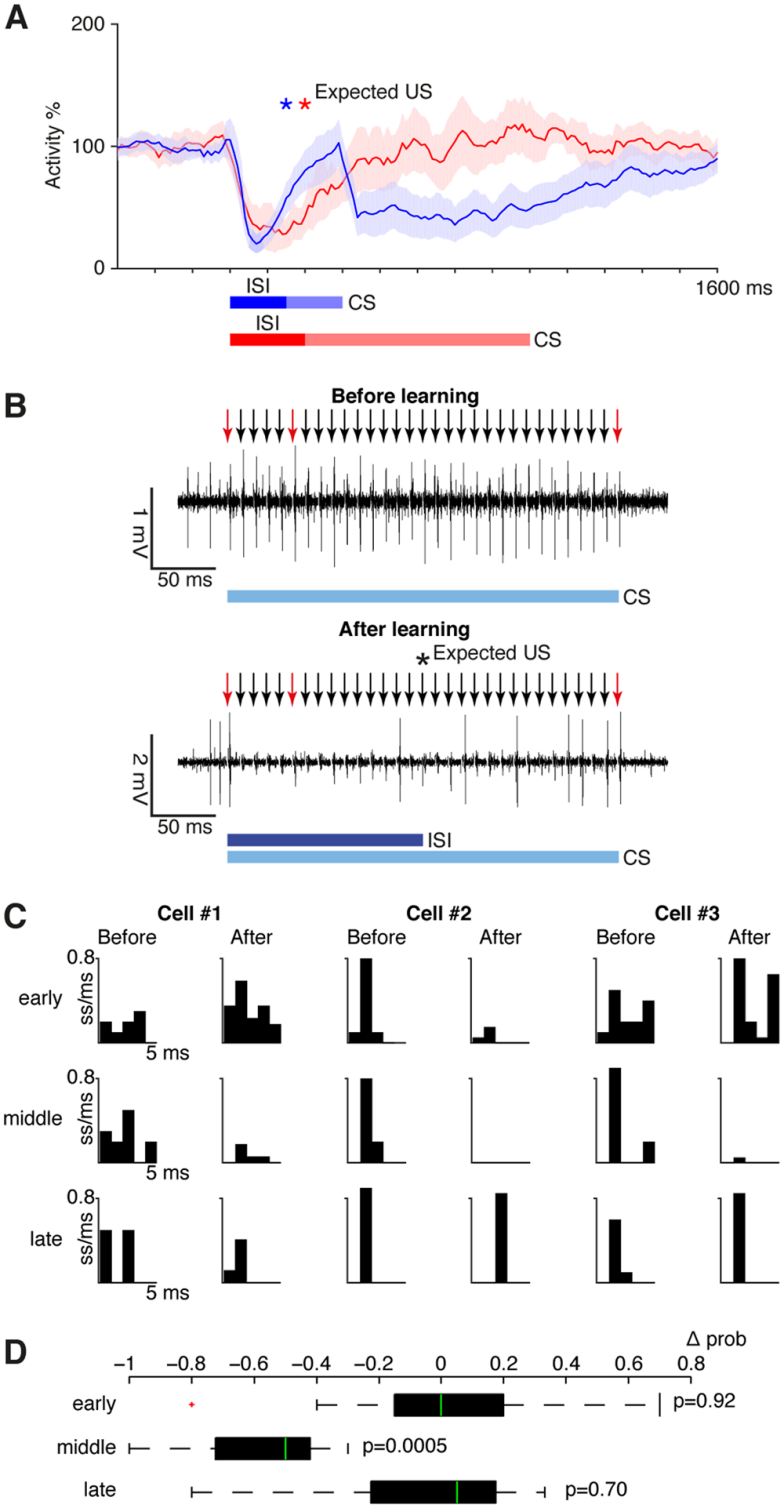
Effects of conditioning on Purkinje cell responses to parallel fibre input. (**A**) Conditioned responses on CS only trials after training with either a 150 ms interstimulus interval (blue, n = 7, CS duration: 300 ms) or a 200 ms interstimulus interval (red, n = 5, CS duration: 800 ms). Traces show smoothed and averaged simple spike activity over 20 trials ± SEM as a percentage of background activity. ISI, interstimulus interval (between onset of the first CS pulse and the expected US). See ref.^8^ for full details on these conditioned responses. (**B**) Sample records of a Purkinje cell’s response to the CS alone before and after learning. Arrows indicate the timing of CS pulses. The red arrows indicate those pulses that were selected for analysis. (**C**) Post stimulus time histograms showing spike probability in three sample cells. These cases illustrate the variability between cells. (**D**) Boxplot showing the average change (Δ prob) in spike probability in all cells for the three selected stimulus impulses (median, 75th and 25th percentiles, maximum and minimum data points not considered outliers and one outlier, defined as 1.5 quartile ranges below the first quartile, marked as ‘+’). P values indicate Wilcoxon matched-pairs signed rank tests mentioned in text.

Twelve Purkinje cells were trained with a CS that consisted of a 100 Hz train of stimulus pulses to the pfs followed by a US consisting of direct climbing fibre stimulation as described previously^8^ and as illustrated in Fig. 1A. To enable analysis of spike probability both before and after the expected US, this is the subset of cells in ref.^8^ where the CS continued beyond the US. In 7/12 cells the CS lasted 300 ms and the US was delivered at 150 ms. In 5/12 cells the CS lasted 800 ms and the US was delivered at 200 ms. In every case, this training protocol resulted in a typical Purkinje cell CR, that is a strong suppression of simple spike firing that reaches a maximum just before the expected US onset and ending shortly after the US, even though the CS continued for an additional 150 or 600 ms (Fig. 2A).

From the CS train, we selected three individual stimuli and determined the probability of a spike response 1–4 ms after each stimulus pulse over 10–20 trials. For comparison, we selected the first stimulus pulse (‘early’), the pulse delivered 100 ms before the US (‘middle’) and the pulse delivered 150 ms after the US (‘late’).

Although there was some variability between cells, the main results were clear (Fig. 2B–D). The average probability of spike responses of the Purkinje cell to the ‘early’ and ‘late’ pf stimuli were virtually identical before and after acquisition of the CR (‘early’: 0.42 +/− 0.09 SEM versus 0.41 +/− 0.13, ‘late’: 0.58 +/− 0.08 versus 0.49 +/− 0.09). A Wilcoxon matched-pairs signed rank test indicated no difference for either the ‘early’ impulse (W = 3; p = 0.92) or the ‘late’ impulse (W = 10; p = 0.70). Only the spike probability to an individual pf stimulus *during* the CR changed (0.69 +/− 0.07 versus 0.11 +/− 0.05, W = 78, p = 0.0005).

## Discussion

The result show that, although the Purkinje cells exhibited clear conditioned pause responses, the conditioning protocol did not cause any measurable depression of the pf/Pc synapses.

It might be suggested that the reason that there was no LTD of the pf/Pc synapses activated by the first pf pulse, was that this set of synapses was activated too far ahead of the climbing fibre US, whereas later pf input, generated by the indirect loops, would be closer in time to the US. This suggestion is contradicted by the LTD literature, according to which the 150 ms CS–US interval employed in our experiments is well within the range for significant LTD^7^. Furthermore, as the CS is a direct train of repetitive stimuli to the same parallel fibers, the set of pf/Pc synapses activated by the first stimulus pulse would also be activated by all the subsequent pulses, and most of them would therefore be closer in time to the US. A substantial proportion of the synapses should therefore have undergone LTD and some degree of reduced spiking probability should have occurred. The fact that hundreds of CS-US presentations did not cause any measurable decrease at all in the probability of a spike after the first CS pulse, is strong evidence that no LTD occurred.

LTD can remove excitation but it cannot by itself suppress Purkinje cell firing below its resting level. It can only do so in combination with inhibitory input^7,8,13^. The present finding is consistent with recent reports suggesting that the Purkinje cell CR is generated by an intrinsic mechanism that does not involve the inhibitory interneurons^8,12,13^ so LTD is not needed.

Although we agree with most investigators that LTD probably has an important role in other forms of motor learning, the present results clearly support the conclusion, that a classical conditioning protocol does not cause any significant LTD and that, therefore, the learning of the Purkinje cell CR in classical conditioning is not due to LTD but involves a different kind of learning mechanisml^7,8,13^.
